# Cu(II) and Mn(II) Anchored on Functionalized Mesoporous Silica with Schiff Bases: Effects of Supports and Metal–Ligand Interactions on Catalytic Activity

**DOI:** 10.3390/nano13121884

**Published:** 2023-06-19

**Authors:** Mihaela Mureseanu, Mihaela Filip, Irina Bleotu, Cezar Ionut Spinu, Alexandru Horia Marin, Iulia Matei, Viorica Parvulescu

**Affiliations:** 1 Department of Chemistry, Faculty of Sciences, University of Craiova, Calea Bucuresti, 107I, 200478 Craiova, Romania; georgi_irina@yahoo.com (I.B.); cezar.spinu@edu.ucv.ro (C.I.S.); 2 “IlieMurgulescu” Institute of Physical Chemistry, Romanian Academy, Splaiul Independentei 202, 060021 Bucharest, Romania; mihaela_burcin@yahoo.com (M.F.); iulia.matei@yahoo.com (I.M.); vpirvulescu@icf.ro (V.P.); 3 Ken and Mary Alice Lindquist Department of Nuclear Engineering, Penn State University, University Park, State College, PA 16802, USA; marin.alexandru.horia@gmail.com; 4 Surface Analysis Laboratory, Institute for Nuclear Research Pitesti, 115400 Mioveni, Romania

**Keywords:** supported Schiff base, metal complexes, functionalized mesoporous silica, cyclohexene oxidation, alcohol oxidation

## Abstract

New series of Cu(II) and Mn(II) complexes with Schiff base ligands derived from 2-furylmethylketone (Met), 2-furaldehyde (Fur), and 2-hydroxyacetopheneone (Hyd) have been synthesized in situ on SBA-15-NH_2_, MCM-48-NH_2_, and MCM-41-NH_2_ functionalized supports. The hybrid materials were characterized by X-ray diffraction, nitrogen adsorption–desorption, SEM and TEM microscopy, TG analysis, and AAS, FTIR, EPR, and XPS spectroscopies. Catalytic performances were tested in oxidation with the hydrogen peroxide of cyclohexene and of different aromatic and aliphatic alcohols (benzyl alcohol, 2-methylpropan-1-ol, and 1-buten-3-ol). The catalytic activity was correlated with the type of mesoporous silica support, ligand, and metal–ligand interactions. The best catalytic activity of all tested hybrid materials was obtained in the oxidation of cyclohexene on SBA-15-NH_2_-MetMn as a heterogeneous catalyst. No leaching was evidenced for Cu and Mn complexes, and the Cu catalysts were more stable due to a more covalent interaction of the metallic ions with the immobilized ligands.

## 1. Introduction

Catalytic oxidation is an important research area due to the variety of its applications for chemical synthesis and environmental protection. Many transition metal complexes were used as catalysts for oxidation reactions by using several oxidants, such as oxygen, hydrogen peroxide, and tert-butyl hydroperoxide (TBHP) [1,2]. The oxidation of cyclohexene has attracted a great deal of attention lately, mainly due to the oxidation products of cyclohexene and the derivatives that provide strongly reactive carbonyl groups in the cycloaddition reactions [3,4,5,6,7,8]. Furthermore, the oxidation of alcohols to the corresponding aldehydes, ketones, and carboxylic acids is one of the important reactions in organic chemistry, especially in industrial synthetic chemistry [9,10,11,12,13,14]. Transition metal Schiff base complexes, as inorganic mimics of enzymes [15,16,17,18], have been extensively studied due to their potential use as catalysts in a wide range of oxidation reactions [19,20,21,22,23,24] and have the advantage of being prepared using simple and cheap methods. The activity of these complexes varies with the type of ligand, coordination sites, and metal ion [25]. Although the homogeneous catalysts show high activity and selectivity, several technical impediments were encountered in their industrial applications, such as difficult separation from reaction products and the deactivation of active sites by self-aggregation. In order to minimize these disadvantages, the heterogenization of homogeneous catalysts has emerged as a focus of research. Several polymer and inorganic oxide-anchored complexes with catalytic activities as good as those of homogeneous complexes have been developed [26,27,28,29,30]. The physical immobilization of transition metal complexes on such supports leads to systems that are susceptible to leaching. More stable catalysts result from a covalent attachment to a functionalized support [31]. Therefore, the covalent anchoring of Schiff base complexes onto a functionalized siliceous mesoporous material with large pore diameters seems to be promising, especially for catalytic applications [32,33,34]. Ordered mesoporous silica are interesting solid supports due to their uniform and large pores, tunable pore sizes, high-surface areas, defined surface acidity, excellent mechanical stability, and high concentration of surface Si–OH groups for the binding of the catalyst active sites [35,36,37]. In particular, MCM-41 (2d hexagonal p6mm), MCM-48 (3d cubic Ia3d), and SBA-15 (large-pore 2d hexagonal p6mm) mesoporous silica are used as supports with a high specific surface area (600–1000 m^2^g^−1^) and narrow pore size distribution (pore diameter ~ 3–10 nm) [38]. These materials present the advantage of easy functionalization with organic groups at the pore–wall interface [39,40], which are used thereafter to immobilize ligands or their complexes with various metal ions. These catalysts combine both the characteristics of their support, such as a controllable pore diameter, large surface area, ordered porous structure, and electrostatic potential, with the electronic and stereo-chemical properties of the metal complex. The porous inorganic host is supposed to allow the right steric configuration and orientation of the metal complex, and so the direct access of the substrate molecules to the active site (the metal center) is regulated. Moreover, the density of surface hydroxyl groups and their activity varies with the synthesis conditions of the mesoporous silica, thus determining the degree of the surface functionalization, respectively, of the properties of the obtained catalysts. The effect of mesoporous silica supports on the covalent attachment of Schiff base complexes with transition metals is still a debated issue. Up to now, Cu(II) complexes, synthesized by other methods, have been used as catalysts in olefin and alcohol oxidation reactions in homogeneous and heterogeneous mediums [41,42,43,44]. It is known that Mn compounds that contain chelating N-ligands are among the most active catalysts in hydrocarbon oxidations [20,26]. Thus, Mn(II) and Mn(III) immobilized complexes were used as efficient and highly selective heterogeneous catalysts in alkene epoxidation [22,26,45] or alcohols oxidation [46]. There are reports on other metal complexes obtained in situ from Schiff base ligands derived from amino-functionalized silica and an aldehyde that were used as active catalysts, but only one of them was tested in oxidation reactions [47]. In this context, the synthesis of new hybrid catalysts based on mesoporous silica–metal complexes is expected to lead to better oxidation catalysts, due to a larger number of active sites, more facile substrate accessibility, and an improvement in diffusional problems.

In this paper, we report the immobilization of Cu(II) and Mn(II) complexes with Schiff base ligands obtained in situ from 2-furaldehyde, 2-furylmethylketone, and 2-hydroxyacetophenone covalently attached to amino-functionalized SBA-15, MCM-41, and MCM-48 materials. These new hybrid catalysts were subsequently utilized in the oxidation reaction of cyclohexene and of different aromatic and aliphatic alcohols (benzyl alcohol, 1-buten-3-ol, 2-methylpropan-1-ol) with H_2_O_2_. Regarding the ligands used in this study, we mention that one of the aldehydes, 2-furaldehyde, was chemically immobilized onto silica gel modified with 3-aminoproyltrimethoxysilane and tested as an adsorbent for Cu(II) and Ni(II) from aqueous solutions [48].

The aim of this study was to evidence the effects of mesoporous silica supports, the nature of the ligand and the metallic ion, on the catalytic activity of the obtained hybrid materials in the studied oxidation reactions. 

## 2. Materials and Methods

### 2.1. Preparation of Functionalized MCM-41, MCM-48, and SBA-15 Silica

A highly ordered hexagonal siliceous MCM-41 was prepared as described in the literature [49]. The resulting gel with the molar composition: 1.00 SiO_2_:0.1 CTABr:25 H_2_O:0.25 NaOH was hydrothermally treated in a Teflon-lined autoclave at 403 K for 24 h. The solid was filtered, washed with deionized water, and dried in air at 353–373 K for 12 h. The as-synthesized MCM-41 was calcined at 823 K under air flow for 6 h in order to remove the template. 

MCM-48 mesoporous silica with the molar composition 1.00 SiO_2_:0.175 CTABr:120 H_2_O:0.38 NaOH was synthesized according to a procedure detailed in the literature [50]. The resulting gel was hydrothermally treated in a Teflon-lined autoclave at 423 K for 15 h. The as-synthesized MCM-48 was washed with water until it reached a neutral pH, dried in air at 353 K, and calcined at 823 K for 8 h in airflow.

SBA-15 material was synthesized as described by Zhao et al. [51]. The gel with the molar composition 1.00 SiO_2_:0.015 P123:5 HCl:140 H_2_O was hydrothermally treated in a Teflon-lined autoclave at 403 K for 24 h. The as-synthesized SBA-15 was calcined at 823 K, under airflow, for 8 h.

The organic-functionalized silica supports were obtained using a post-grafting procedure with 3-aminopropyltriethoxysilane (APTES) according to a previously described procedure [52].

### 2.2. Synthesis of Schiff Bases Grafted onto Functionalized Silica

The Schiff bases were synthesized by the in situ condensation of an aldehyde or a ketone onto the amino-functionalized silica supports (MCM-41-NH_2_, MCM-48-NH_2_, SBA-15-NH_2_) as described in the literature [53]. Briefly, 1 g amino-functionalized silica (MCM-41-NH_2_, MCM-48-NH_2_, SBA-15-NH_2_) was activated at 393 K for 3 h. The activated support was refluxed with an excess of 2-furaldehyde (Fur), 2-furylmethylketone (Met), or 2-hydroxyacetophenone (Hyd) in anhydrous ethanol at 333 K, under N_2_ atmosphere, for 24 h. The solids were filtered, washed, and dried in air at 333 K overnight. The pale-yellow products were denoted as: SBA-15 (MCM-41, MCM-48)-NH_2_-Fur, SBA-15 (MCM-41, MCM-48)-NH_2_-Met, and SBA-15 (MCM-41, MCM-48)-NH_2_-Hyd.

### 2.3. Synthesis of Cu(II) and Mn(II) Complexes with Schiff base Ligands

The heterogeneous Cu(II) and Mn(II) complexes were obtained by mixing 0.5 g of (Schiff-base)-grafted silica and 10 mmols of Cu(NO_3_)_2_·3H_2_O or Mn(NO_3_)_2_·6H_2_O in 25 mL of methanol at room temperature for 24 h. The solids were filtered, washed and dried overnight.

### 2.4. Characterization of Materials

Small-angle XRD data were acquired on a Bruker AXS D8 diffractometer by using Cu Kα (λ = 0.1541 nm) radiation and a Ni filter. The samples were scanned in the range 2θ = 0.4–6.0°. N_2_ adsorption/desorption isotherms were measured at 77 K with a Micromeritics ASAP 2010 instrument (Micromeritics, Norcross, GA, USA). Before taking measurements, the samples were outgassed at 323 K for 12 h. The specific surface area was calculated using the BET method and the mesopore volume was determined from the isotherm at the end of the capillary condensation. The pore size distribution was obtained from the desorption branch using the BJH method and the Harkins–Jura standard isotherm. FTIR spectra of all samples were performed in KBr pellets using a Bruker Alpha spectrometer (from Bruker Optics, Leipzing, Germany). Atomic absorption spectroscopy (AAS) measurements were performed on a GBS Avanta spectrometer equipped with multi-element hollow cathode lamps and an air-acetylene burner. The C, H, and N contents were evaluated by combustion on a Fisons EA1108 elemental analysis apparatus (Thermo Fisher, Waltham, MA, USA). Thermogravimetric analysis was carried out in a Setaram thermobalance. XPS analysis was performed on a X-ray photoelectron spectroscopy (XPS) system PHI Quantera SXM (ULTRAVAC-PHI. INC., Chigasaki, Kanagawa, Japan), and EPR spectra were recorded on a JEOL FA 100 spectrometer (JEOL Tokyo, Japan). Chemical microanalyses by scanning electron microscopy (SEM) were performed on a Hitachi S-4500 I scanning microscope (Hitachi, Chiyoda City, Tokyo), and transmission electron microscopy (TEM) characterization was recorded on a JEOL JEM 1200 EX II (JEOL, Tokyo, Japan) operated at 100 kV.

### 2.5. Catalytic Test

The catalytic oxidation reactions were carried out in 5 mL flasks using acetonitrile as a solvent and H_2_O_2_ (30% aqueous) as an oxidant. The amount of catalyst was 30 mg, and the tested substrates were cyclohexene (CH), benzyl alcohol (BzA), 2-methylpropan-1-ol (IzBA), and 1-buten-3-ol (1B3A). The molar ratio substrate/solvent/oxidant was 1/3.8/3. The reaction mixture was heated at 303 K for 5 h with continuous stirring.

The reactants and oxidation products were analyzed using a Thermo DSQ Single Quadrupole GC/MS with Thermo Triplus autosamplers (Thermo Fisher Scientific USA) with capillary column TR-5MS (30 m × 0.25 ID × 0.25 μm film thickness). Samples were filtered before analysis. The catalytic activity was expressed as substrate conversion and also as a turnover frequency (TOF) in order to highlight the kinetic effects on the catalytic activity of the different types of mesoporous silica supports. TOF, in h^−1^, is calculated as moles of the substrate converted per moles of metal ion per hour.

### 2.6. Leaching Test and Reusability

The active compound leaching during the reaction was verified for SBA-15-NH_2_MetMn and SBA-15-NH_2_-MetCu samples using the hot filtration test and the resubmission of filtrates to reaction conditions in order to measure their residual activity.

The reusability of these two catalysts was checked in the oxidation of CH for 5 consecutive runs. The copper and manganese content in the recovered catalysts was determined by AAS after each run.

## 3. Results and Discussion

New heterogeneous catalysts were obtained and tested for their oxidative activity.

**Scheme 1:**
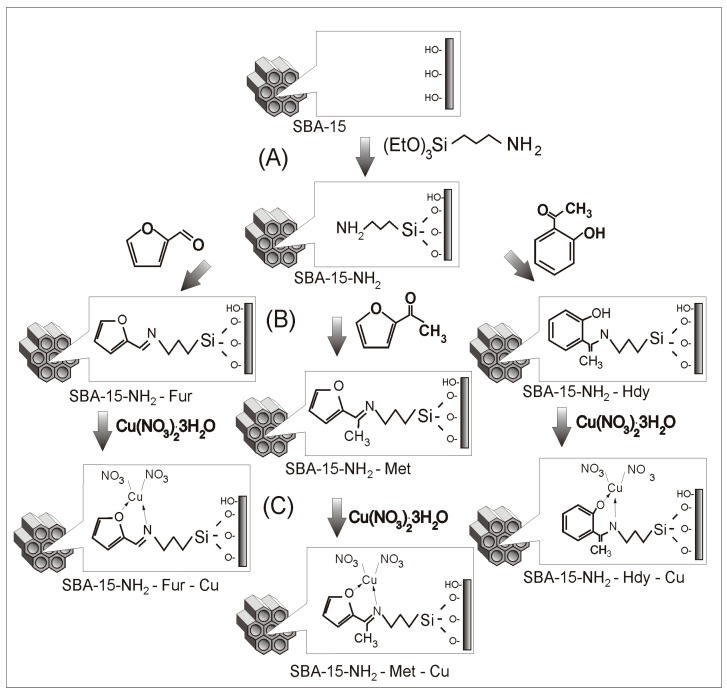
Synthesis of Cu(II) complexes with Schiff base ligands derived from 2-furaldehyde, 2-furylmethylketone, and 2-hydroxyacetophenone on SBA-15 silica mesoporous support.

The supported Cu(II) and Mn(II) complexes with Schiff base ligands were synthesized in three steps: (i) post-synthesis functionalization of MCM-41, MCM-48, and SBA-15 mesoporous silica supports with APTES; (ii) condensation of surface NH_2_ functional groups with 2-furylmethylketone, 2-furaldehyde, or 2-hydroxyacetopheneone to form the corresponding Schiff base; and (iii) in situ Cu(II) or Mn(II) coordination with Schiff base ligands to obtain the supported metal complexes (Scheme 1). We mention that the presented structure of Cu(II) and Mn(II) complexes immobilized on mesoporous silica support is in agreement with the structure of other heterogeneous metal complexes with bidentate Schiff base ligands [29] and is used for representation purposes only.

### 3.1. Structure, Texture, and Morphology of the Support Materials

The structural, textural, and morphological characteristics of the obtained silica supports are typically for MCM-41, MCM-48, and SBA-15 mesoporous silica.

**Figure 1:**
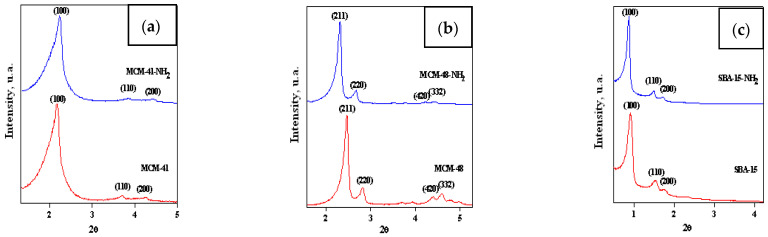
XRD patterns of (**a**) MCM-41, (**b**) MCM-48, and (**c**) SBA-15 before and after amino-functionalization.

The small angle XRD patterns, before and after functionalization with amino groups, show the characteristic peaks for these materials (Figure 1).

**Figure 2:**
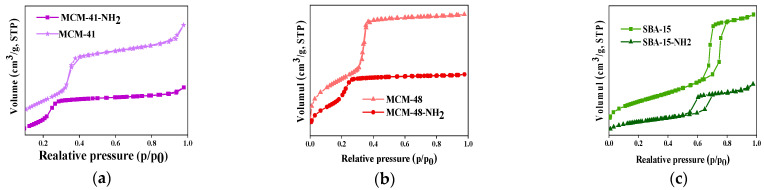
N_2_ adsorption–desorption isotherms of MCM-41 (**a**), MCM-48 (**b**), and SBA-15 (**c**) samples before and after their functionalization.

XRD patterns of the MCM-41 material (Figure 1a) emphasize an ordered structure with three diffraction peaks (100, 110, and 200), indicating ordered two-dimensional hexagonal symmetry. Structural analysis of the MCM-48 support reveals that it has a cubic ordered structure with a three-dimensional arrangement characteristic for this type of silica (Figure 1b). SBA-15 material shows three diffraction peaks (Figure 1c) associated with the typical two-dimensional hexagonal symmetry (P6mm). A slight decrease in the well-resolved peak intensity in the range of 0.5–5° after functionalization was evidenced for all three samples. This indicates a low variation in the inorganic wall structure after the functionalization with the amino groups. A similar effect can be observed for the porous structure parameters and wall thickness values of all the mesoporous silica supports (Table 1). The N_2_ adsorption/desorption isotherms of modified mesoporous silica confirmed that the channels of the support remained accessible. All amino-functionalized materials maintained the characteristics of type IV isotherms (Figure 2) and showed a uniform pore size distribution in the mesoporous region, similar to the genuine support. However, the pore diameter and pore volume decreased compared to the pure siliceous material.

A pronounced decrease in the BET surface area and of the pore volume of the hybrid materials was evidenced, as well as an increase in the wall thickness, due to the increase in the organic group content (Table 1).

**Figure 3:**
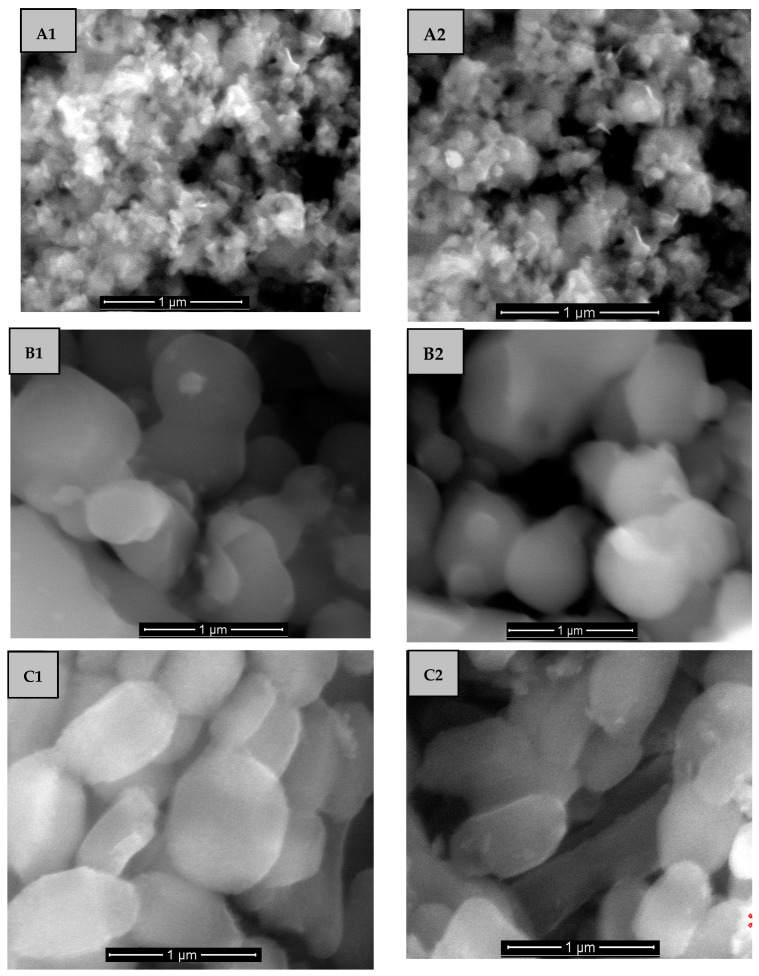
SEM images of (**A1**)-MCM-41, (**A2**) MCM-41-MetCu; (**B1**)-MCM-48, (**B2**) MCM-49-Met-Cu, (**C1**)-SBA-15, and (**C2**) SBA-150MetCu samples.

SEM images (Figure 3) highlight the spherical morphology of MCM-41 and MCM-48 supports and rod-like morphology typical of SBA-15 materials. The nature of the complexes does not have any significant impact on the morphology.

**Figure 4:**
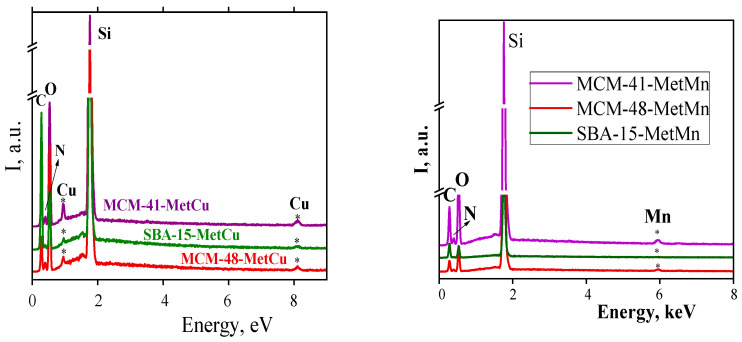
SEM-EDAX results for the supported Cu and Mn complexes.

**Figure 5:**
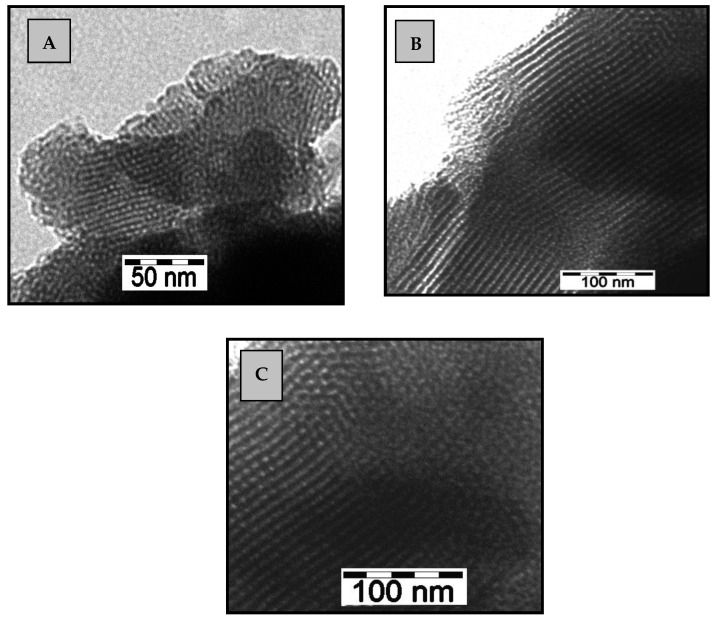
TEM Images of (**A**) MCM-41, (**B**) MCM-48, and (**C**) SBA-15 mesoporous silica supports.

The composition of these immobilized Cu complexes was evidenced by SEM-EDAX, comparatively with that of Mn complexes (Figure 4). Detailed structural characterization has been conducted for MCM-41, MCM-48, and SBA-15 mesoporous silica using TEM microscopy (Figure 5). In all these cases, the porous structure was clearly visualized. TEM images show for MCM-41 samples (Figure 5A) a uniform array of pores with a hexagonally arrangement and a highly ordered three-dimensional pore arrangement for MCM-48 materials (Figure 5B). The TEM micrograph of SBA-15 (Figure 5C) shows the hexagonal array of uniform channels. TEM characterization is in good agreement with the XRD and N_2_ adsorption–desorption results discussed above. The effect of functionalization on the structure and morphology of the porous support was very low.

On the surface of MCM-41, MCM-48, and SBA-15 mesoporous silica, there are a lot of silanol groups that can serve as sites for the incorporation of the aminopropyl groups. The literature [54] presents a higher concentration of silanol groups for SBA-15 and MCM-48 (~13 mmol/m^2^) compared to MCM-41 (~2 mmol/m^2^). Considering the values of the surface area presented in Table 1, the variation in the silanol groups density on the support surface is as follows: Si-OH_MCM-41_ < Si-OH_SBA-15_ < Si-OH_MCM-48_.

### 3.2. Immobilization of the Metal Complexes

**Figure 6:**
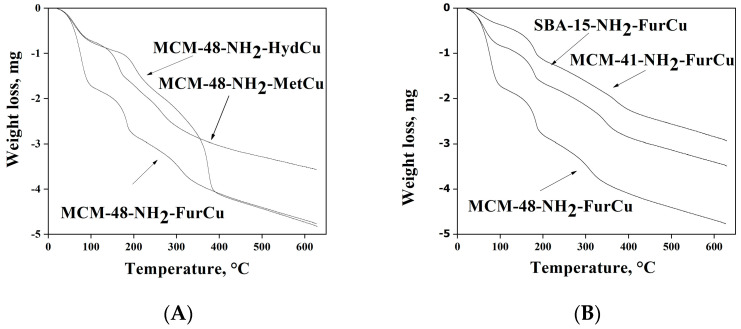
Effect of ligand (**A**) and support type (**B**) on mass loss evidenced by TGA analysis.

Thermogravimetric analysis (TGA) of the supported Cu(II) and Mn(II) complexes with Schiff base ligands indicates three major mass losses (Figure 6). For all materials, the mass losses at 100 °C and at around 200 °C were attributed to the solvent elimination, respectively, to the partial decomposition of the metal complexes. The final decomposition of the metal complexes and total organic compound loss started at ~300 °C for the immobilized complexes with 2-furylmethylketone or 2-furaldehyde and at ~400 °C for supported Schiff bases with 2-hydroxyacetophenone (Figure 6A).

The effect of the mesoporous silica support type on the shape of the TG curves was insignificant (Figure 6B). However, the weight loss was significantly influenced by the surface area of the support. Higher weight loss was evidenced for metal complexes immobilized on the MCM-48 supports with the highest surface area. The amount of immobilized ligand (L), immobilization yield (Y_imm._ %), and the metal-to-ligand molar ratio (M/L) values were obtained by elemental analysis and are presented in Table 2. The amount of immobilized ligand was determined from TG curves by subtracting the amount of the aminopropyl group previously grafted on the mesoporous silica surface and compared with the elemental analysis (C%, N%, and H%). There was a good correlation between the results obtained by both methods. The copper and manganese contents of all samples were determined by AA spectroscopy after their dissolution in a 10% HF solution.

The weight loss due to the aminopropyl moiety was 11.61% for the SBA-15, 11.11% for MCM-41, and 13.89% for MCM-48 mesoporous silica, respectively. These values correspond to an aminopropyl density of 3.1 molecules/nm^2^ for SBA-15 silica, 1.7 molecules/nm^2^ for MCM-41, and 1.5 molecules/nm^2^ for MCM-48.

**Figure 7:**
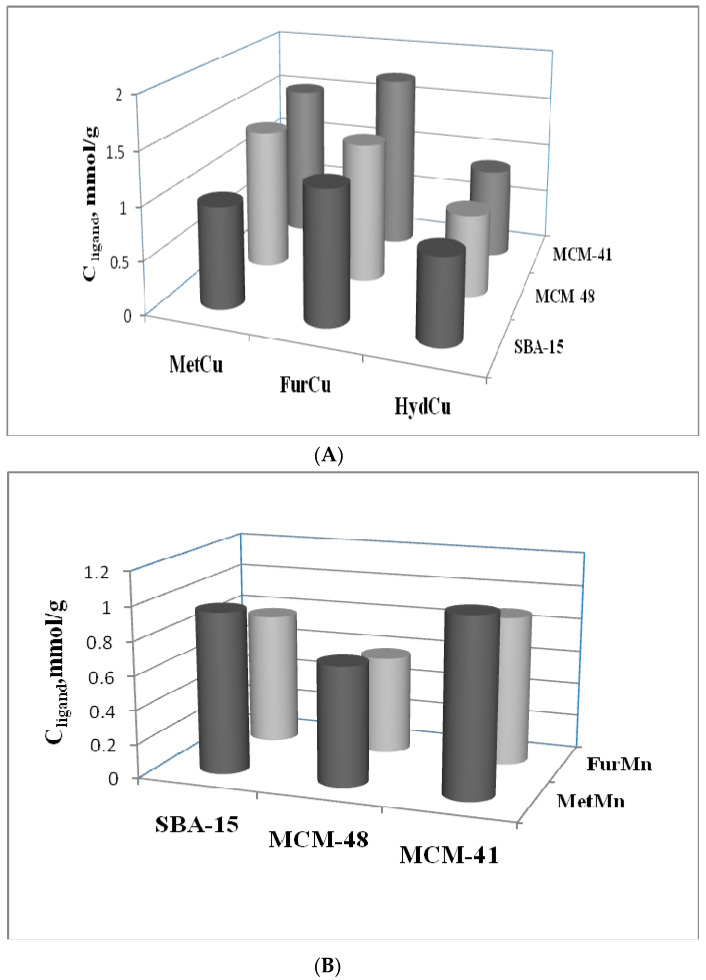
Variation in the ligand concentration on support surface for copper complexes (**A**) and manganese complexes (**B**).

For all three types of mesoporous silica, considering the specific surface area and the concentration of silanol groups on the support surface, we can conclude that the highest degree of silanol participation in the functionalization with amino groups and the most hydrophobic surface was obtained for MCM-41 support. This is in agreement with the results presented in Table 2 and Figure 7A, indicating the best immobilization yield for all the metal complexes with the studied Schiff bases on the MCM-41 support. Even if the density of the –NH_2_ groups was greater in the case of SBA-15 silica than on MCM-41, the more hydrophobic surface of the last support was beneficial to the immobilization of the Schiff base ligand.

The elemental analysis confirmed that between 39 and 89% of the aminopropyl group amount grafted on the silica support surface participated in the Schiff base synthesis by condensation with the corresponding aldehyde or ketone. The ligand density varied between 1.9 and 1.2 molecules/nm^2^ for SBA-15, between 1.5 and 0.9 molecules/nm^2^ for MCM-41, and between 0.8 and 0.4 molecules/nm^2^ for MCM-48, respectively. The results presented in Table 2 were obtained for the supported metal complexes after removal, by washing, of the species that are weakly bound on the surface. In these conditions, we consider that only the unreacted aminopropyl groups and the newly formed Schiff base ligands were presented on the silica support surface. Furthermore, not all of the Schiff base ligands have been involved in the complexation reaction with the copper and manganese cations, respectively.

The metal-to-ligand molar ratio varied between 1/3 and 1/6 for copper complexes and between 1/5 and 1/9 for manganese complexes, respectively. Metal ion complexation by the unreacted aminopropyl groups was also possible. It is clear that the complexation reaction is much more favorable for copper ions than for manganese ions (Figure 7A,B). Figure 7B presents the variation in the ligand concentration with the silica support type for MetMn and FurMn complexes, respectively. For all three types of mesoporous silica, a lower ligand amount was obtained for the Schiff base derived from 2-furaldehyde. Even if for MetMn complex the amount of immobilized ligand was greater, the metal content of all the samples was almost the same as for FurMn.

Although the ligand amount was initially the same, the amount of copper and manganese complexes with the same ligand changed after complexation. A greater amount of Cu(II) could stabilize the corresponding complex, which explains the greater amount of ligand determined from elemental and TG analysis for the samples of immobilized copper complexes.

### 3.3. Spectroscopic Analysis of the Supported Metal Complexes

**Figure 8:**
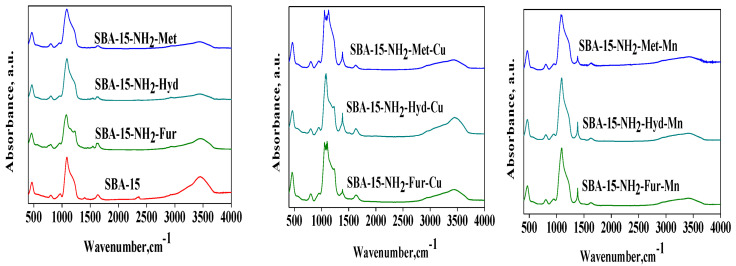
FTIR spectra of the support and of the metal complexes with Schiff base ligands immobilized onto SBA-15 mesoporous silica.

The FTIR spectra for the Schiff bases obtained in situ by grafting 2-furylmethylketone, 2-furaldehyde, or 2-hydroxyacetophenone onto the amino-functionalized silica (Figure 8) and the corresponding Cu(II) and Mn(II) complexes were registered in the 4000–400 cm^−1^ range and were used to characterize the support and to confirm the complex formation on the solid samples after modification.

The spectrum of the unmodified SBA-15 samples and the spectra of all the modified materials are dominated by strong bands characteristic of the silica matrix, indicating that the support framework remained unchanged. The FTIR spectra of the free and amino-functionalized supports present bands that could be attributed to the vibration modes of the silica host matrix [55]. Symmetrical stretching vibrations of Si-O-Si bonds are observed around 790 cm^−1^. The bands at 460 cm^−1^ could be assigned to the bending vibrations of the associated Si-O-Si bonds. Bands at around 1080 cm^−1^ could be due to antisymmetric stretching vibrations of Si-O-Si, overlapped to Si-O-C, C-O-C, and Si-C bond vibrations [56]. The existence of silanol bands at 980 cm^−1^ could be referred to as stretching vibrations of free silanol (Si-OH) groups on the surface of the amorphous solid samples. The stretching vibrations of C-O bonds are also in this range [57]. The broad band at around 3450 cm^−1^ corresponds to water molecules bonded to each other and to Si-OH groups by hydrogen bonds. The medium intensity bands at 2922 and 2885 cm^−1^ correspond to the asymmetric and symmetric vibration of –CH_2_- from the APTES functionalization agent [58]. In the spectra of Schiff base functionalized silica, the bands at around 1620 cm^−1^ could be related to the bending vibrations of the O-H bonds in OH groups, overlapped with the characteristic vibration bands for C=N groups. The condensation reaction between the primary amines on the surface and the carbonyl functionalities can be detected by the appearance of imine (C=N) bands at 1645 cm^−1^ for SBA-15-NH_2_- Fur, at 1630 cm^−1^ for SBA-15-NH_2_-Hyd, and at 1616 cm^−1^ for SBA-15-NH_2_-Met, respectively. The FTIR bands of the azomethine group from Schiff bases complexes shifted to a lower frequency (10–15 cm^−1^), which confirms the coordination of nitrogen from this group with the metal atoms [59]. Furthermore, the appearance of the new band at around 1385 cm^−1^ in the spectra of Schiff base complexes could be the result of a red shift of the C=N stretching vibration due to the coordination of copper ions [60]. For all the immobilized complexes, the vibration bands in the low frequencies range (300–800 cm^−1^) attributed to ν_M-O_ and ν_M-N_ are present, but they overlapped with those characteristics for the silica skeleton.

**Figure 9:**
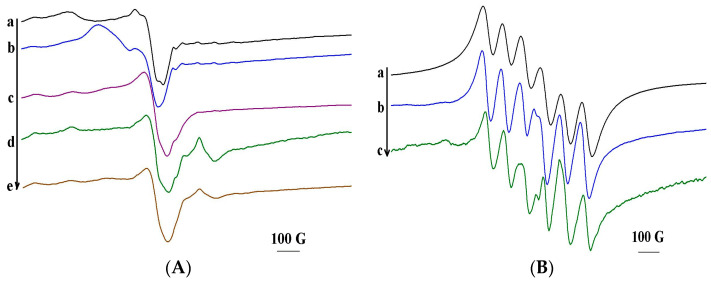
The EPR spectra of copper complexes (**A**) MCM-41-NH_2_-Fur Cu (a), MCM-41-NH_2_-MetCu (b), MCM-48-NH_2_-HydCu (c), SBA-15-NH_2_-MetCu (d), and SBA15-NH_2_-FurCu (e) and (**B**) MCM41-NH_2_-FurMn (a), MCM-48-NH_2_-MetMn (b), and MCM-41-NH_2_-HydMn (c).

The EPR spectra of the copper and manganese complexes immobilized on silica materials, recorded at room temperature, are shown in Figure 9A,B, respectively.

The EPR features of copper complexes are more sensitive to the structural particularities of the functionalized mesoporous silica. These results are somehow expected as the metal–ligand bonds are stronger in complexes with copper than with manganese ions. The EPR parameters obtained by simulation of the experimental spectra of the complexes immobilized in mesoporous materials are summarized in Table 3.

Different g_┴_ and A_║_ parameters in the EPR spectra of Cu(II) complexes were attributed to structural changes in the ligands, different distances between adjacent ligands, and the change in the symmetry around the metal center [61]. The variation in *g*_║_ values from 2.22 to 2.34 indicatesthe presence of a significant covalent bonding in these complexes [62,63]. Thus, values of the covalency parameter (*α*^2^) reveal that Cu(II) complexes have between a 44 and 80% covalent character in the environment of the studied ligands (Table 3) [64]. The trend g_║_ > g_┴_ > 2.0023, observed for copper complexes, shows the localization of the unpaired electron in the metal dx^2^ − y^2^ orbital, with elongated tetragonal symmetry. The EPR spectra of the Cu(II) complexes supported on MCM-41 show an axial anisotropy, while those of MCM-48-NH_2_-HydCu, SBA-15-NH_2_-MetCu, and SBA15-NH_2_-FurCu complexes indicate orthorhombic geometry and hyperfine splitting due to the interaction between Cu nuclear spin and electron spin. 

The values of the geometric parameter G are calculated using the following equation, G = (*g*_║_ − 2.0023)/(*g*_┴_ − 2.0023) [65], where they are higher than 4.0 for all the Cu(II) complexes immobilized, except MCM-41-NH_2_-MetCu. For this last sample, spin–exchange interaction is negligible, the local tetragonal axes are parallel aligned or only slightly misaligned, and ligands can be seen as having a weak field [65,66]. Furthermore, the G factor is lower than 4.0 for this sample, proving a significant exchange coupling with appreciable misalignment.

The EPR spectra of the immobilized Mn(II) complexes (Figure 9B) show six hyperfine lines, with g values very close to that of the free electron, characteristic of oxygen-coordinated Mn(II) ion in disordered systems [67]. The simulation of the EPR spectra recorded for solid Mn complexes indicated an anisotropy of the hyperfine splitting characteristic of a 5/2 spin state. The overall broadening of the spectra for the Mn(II) complexes was similar, which suggests that the Mn(II)–ligand interactions are weaker and bonds are more ionic than for the Cu(II) complexes.

**Figure 10:**
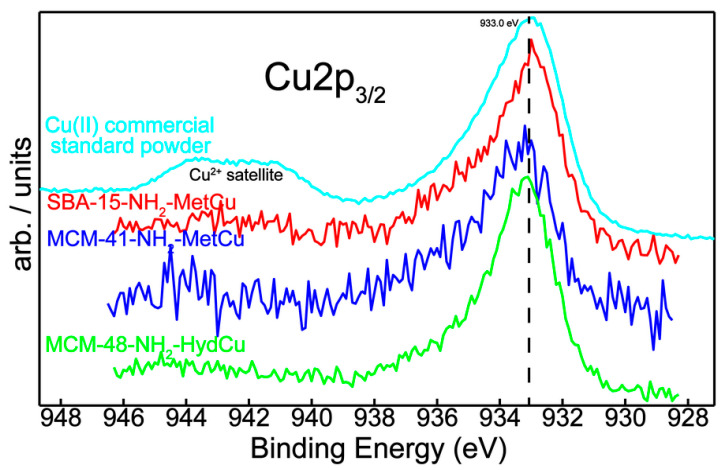
Cu2p_3/2_ XPS superimposed spectra for Cu^2+^ commercial standard powder, SBA-15-NH_2_-MetCu, MCM-48-NH_2_-HydCu, and MCM-41-NH_2_-MetCu samples.

**Figure 11:**
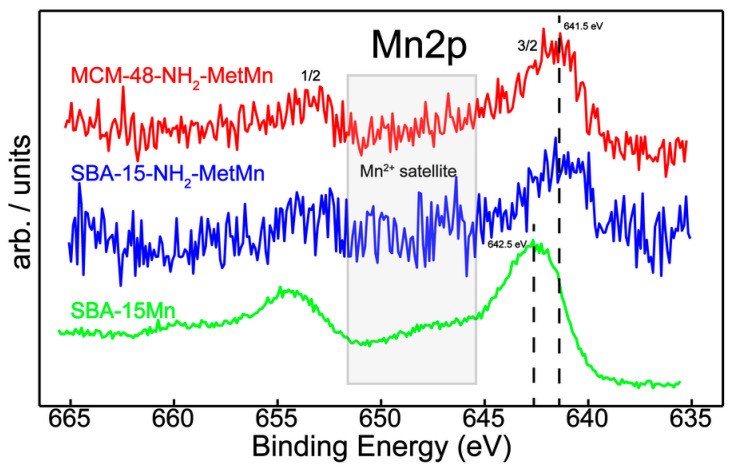
Mn2p XPS superimposed spectra for SBA-15Mn, SBA-15-NH_2_-MetMn, and MCM-48-NH_2_-MetMn samples.

The XPS spectra of the immobilized Cu(II) and Mn(II) complexes (Figure 10 and Figure 11) were compared with the standards obtained by the impregnation of mesoporous silica supports with copper or manganese nitrate.

For all the tested supports, the amount of Cu and Mn was greater for the impregnated samples than for those containing immobilized complexes. These results, revealed by XPS analysis, evidenced changes in the distribution of metals on the silica surface and on the functionalized support surface. The XPS spectra of the immobilized complexes show the highest Cu content (~1.0%) for MCM-48-NH_2_-Met Cu (Figure 10), and these results are in agreement with the results presented in Figure 6. The highest concentration of Mn complexes (~0.6%) was obtained for MCM-48 support (Figure 11). Comparing the amount of immobilized complex correlated with the ligand type, a lower concentration of metal has been observed for Hyd-Cu complexes, results that are in agreement with elemental analysis.

Cu-impregnated samples mainly show a Cu2p_3/2_ broad profile with the peak shape and binding energy being characteristic to the Cu(II) species [68]. This was further confirmed by the perfect match with the standard Cu(II) commercial powder (Figure 10). Moreover, the XPS spectra of the immobilized complexes show that N1s binding energy, attributed to –N=C, has a small shift to a higher binding energy (~399.5 eV) when compared to the imine-specific band [69,70]. This positive shift could be the result of an electron density decrease around the N atom due to metal coordination. A similar effect was revealed by the corresponding shift of O1s binding energy to a higher binding energy (~532.5 eV) compared to the O1s band of the furan ring. 

Figure 11 displays the noisy Mn2p spectra for SBA-15-NH_2_-MetMn and MCM-48-NH_2_-MetMn, suggesting a low content of manganese present on the outermost surface layer. The atomic concentrations for SBA-15-NH_2_-MetMn and MCM-48-NH_2_-MetMn samples were found in the range of ~0.5%. The binding energy (642.5 eV) as well as the presence of a shake-up characteristic satellite exhibit the fingerprints for MnO in the SBA-15Mn impregnated sample. The negative shift of Mn2p binding energy for the immobilized complexes compared with the Mn-impregnated sample could be the result of an electron density increase around the metal ion due to an electron transfer from the ligands.

### 3.4. Catalytic Properties

The catalytic activity of Cu(II) and Mn(II) complexes with Schiff base ligands derived from 2-furylmethylketone, 2-furaldehyde, and 2-hydroxyacetophenoneimmobilized into different NH_2_-functionalized mesoporous silica supports was evaluated for the oxidation of cyclohexene (CH) and aromatic or aliphatic alcohols such as benzyl alcohol (BzA), 2-methylpropan-1-ol (IzBA), and 1-buten-3-ol (1B3A). The oxidative catalytic conversion of organic compounds has been conducted in CH_3_CN as a solvent with peroxide hydrogen (30% H_2_O_2_). H_2_O_2_ is the preferred oxidant in these systems since it is highly mobile in the nanopores due to its smaller size. The solvent plays an important and sometimes decisive role in catalytic behavior because it can make uniform different phases, thus promoting mass transportation, and could also change the reaction mechanism by affecting the intermediate species, the catalyst surface properties, and reaction pathways. We have chosen for the catalytic tests CH_3_CN, which is a polar aprotic solvent usually used in hydrocarbon oxidation reactions. A series of blank experiments revealed that each component is essential for an effective catalytic reaction, and the system is relatively unaffected by changing the order of adding components. No oxidation of cyclohexene or alcohols occurred in the absence of the catalyst. 

**Figure 12:**
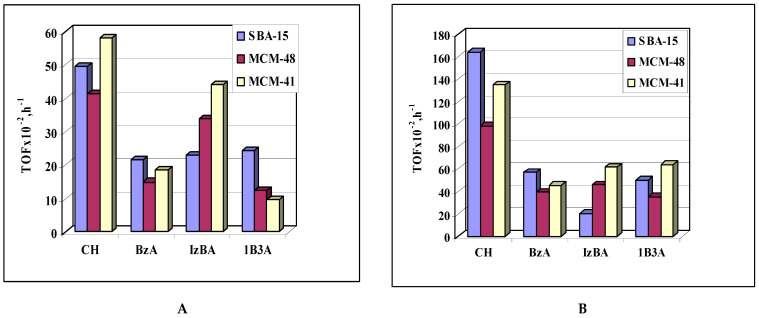
The variation in TOF values for the supported MetCu (**A**) and MetMn (**B**) catalysts in the cyclohexene and alcohols oxidation as a function of the mesoporous silica type.

**Figure 13:**
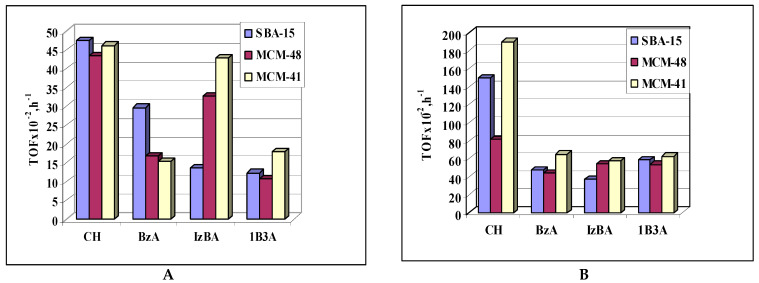
The variation in TOF values for the supported FurCu(**A**) and FurMn(**B**) catalysts in the cyclohexene and alcohols oxidation as a function of the mesoporous silica type.

**Figure 14:**
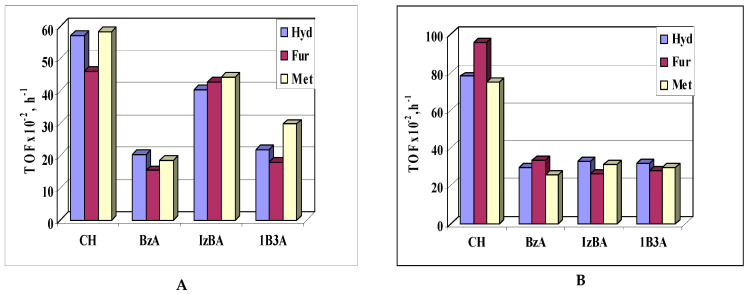
The variation in TOF values for the Cu (**A**) and Mn (**B**) complexes supported on MCM-41 in the cyclohexene and alcohols oxidation as a function of ligand type.

The best activity of the hybrid catalysts was obtained in the oxidation of cyclohexene (Figure 12, Figure 13 and Figure 14, Table 4). Comparing TOF values obtained for copper and manganese complexes with the Schiff base derived from 2-furaldehyde and 2-furylmethylketone immobilized on the mesoporous silica supports (Figure 12 and Figure 13) evidenced the highest activity in the oxidation of CH for MCM-41-NH_2_-MetCu. The copper complexes of Schiff base immobilized into MCM-41 support could present a much more favorable structure and environment for the catalytic reaction, as the EPR parameters also indicated (Table 3).

Thus, the EPR parameter, G, shows a different geometry for these samples, and the geometric arrangement for the FurCu complex is similar to that of copper complexes with a Schiff base supported on other mesoporous silica. These variations are in agreement with the catalytic results.

Even if the covalency parameter (α^2^) of MetCu complexes supported on the MCM-41-NH_2_ sample has a value close to those of the immobilized complex onto SBA-15 and MCM-48 supports, there is a bigger difference between their catalytic activities, indicating that for the two last types of mesoporous silica supports, a more significant effect on the catalytic activity in CH oxidation has the geometric factor. The effect of the covalency factor is more significant in the oxidation of alcohols. For the MetCu complex supported on MCM-41, the order of oxidation activity was CH > IzBA > BzA > 1B3A. A similar effect was evidenced for the FurCu complex in the oxidation of CH, IzBA, and 1B3A. 

**Figure 15:**
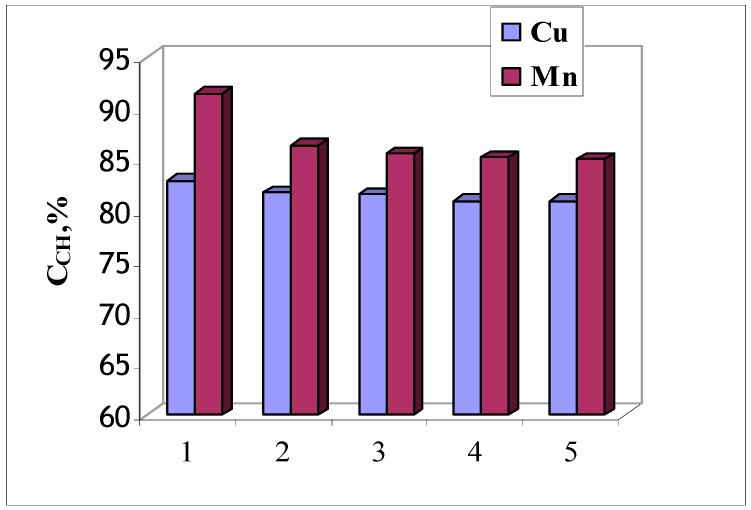
Reusability tests for SBA-15-NH_2_-MetMn and SBA-15-NH_2_-MetCu in CH oxidation reaction.

The variation in the TOF values for these supported catalysts is consistent with the variation in the ligand content on the surface of SBA-15, MCM-48, and MCM-41 silica supports (Figure 7). These results argue that the main effect of the silanol concentration is on Schiff base formation, complexation, and substrate accessibility for the chemical reaction. The influence of support and ligand type on catalytic activity was insignificant for the immobilized Mn complexes, except for the reaction of CH oxidation (Figure 13B, Figure 14B, and Figure 15). However, the highest catalytic activity was obtained for Mn complexes, especially for cyclohexene oxidation, in conditions of a significantly lower Mn/L ratio (Table 2).

Smaller amounts of manganese, EPR spectra, and leaching tests confirm the weaker interaction of Mn ions with ligands and the fact that their bonds are more ionic than in the case of Cu complexes. There is no correlation between the manganese amount and the nature of its interactions with the ligands in the complex. Most probably, for the Mn(II) complexes, higher dispersion of the metallic active sites is more important than their concentration.

With respect to the ligand effect on the catalytic activity, the results indicate the next general order of activity depending on ligand type: Met > Fur > Hyd either for Cu(II) or Mn(II) complexes. One exception was observed for the manganese complex supported on MCM-41 (Figure 14), where the activity of the FurMn complex was slightly higher than that of MetMn.

For all the tested catalysts, the relatively high TOF values proved that the mesoporous silica support offers high substrate accessibility and an improvement in the diffusional problems. The most important parameters for better catalytic activity are a high dispersion of the active site (especially for Mn) and the right steric configuration and orientation of the metal complexes for the direct access of the substrate to the metallic center. 

For the Cu and Mn complexes with Schiff bases that we have tested, the best support was the MCM-41 mesoporous silica. The MCM-41 support, due to its hydrophobic surface, allowed the greatest amount of metal complexes to immobilize (Table 2, Figure 4); consequently, more catalytically active centers are available for the tested oxidation reaction.

Among olefins, cyclohexene is one of the most studied substrates in oxidation reactions. In cyclohexene oxidation, two reactions can take place: oxidation in the allylic position and breakdown of the C=C double bond. In the second one, cyclohexene oxide, an interesting monomer for its applications [71,72], could be obtained, or cyclohexane 1,2 diol after oxide hydrolysis. When studying the CH oxidation by using Mn(II) and Cu(II) complexes immobilized into mesoporous silica supports, both reactions take place, but the main followed route was oxidation in allylic position. Table 4 shows a higher selectivity for 2-cyclohexen-one and a very low selectivity for cyclohexene oxide. The presence of 1,2-cyclohexendiol evidence the effect of H_2_O_2_ aqueous solution on epoxide hydrolysis.

No significant effect on selectivity was observed for the type of mesoporous support. For all the catalysts, the highest selectivity was for 2-cyclohexen-1-one. However, a variation in the cyclohexene oxide selectivity was evidenced. A higher selectivity (around 2%) was obtained for the MCM-48 support and HydCu complex.

The main product in the oxidation of benzyl alcohol was benzaldehyde, and for the oxidation of 2 methylpropan-1-ol, it was 2 methylpropanal. The oxidation of 1-butene-3-ol, an alcohol possessing both the hydroxyl and olefinic functional group, led to 1 buten-3-one as the main product. Chemoselective oxidation of this unsaturated alcohol can occur via two possible reaction pathways: the epoxidation of C=C bound and the allylic oxidation of alcohol to aldehyde [73]. Usually, epoxidation is the aim of 1-butene-3-ol oxidation because the epoxide allows many applications, such as the synthesis of 1,2-epoxybutane-3-ol. Unfortunately, this reaction requires special experimental conditions such as higher temperature (100–120 °C) and pressure [74]. In the conditions of our reactions, only 1-butene-3-one was identified.

When comparing the oxidative activity of these new catalysts with other heterogeneous metal complexes, both the substrate conversion and TOF values are very good. For example, unsubstituted and tertiary-butyl substituted salicylaldimine complexes of Cu(II) and Co(II) were immobilized on silica supports (MCM-41, SBA-15, and Davisil 710) and tested as a catalyst for cyclohexene oxidation using hydrogen peroxide as an oxidant under an oxygen atmosphere [75]. All catalysts were active in the tested reaction, and the conversions varied between 41% and 84% depending on the nature of the catalyst. A copper(II) ethyl acetonate precursor was immobilized onto organofunctionalized MCM-41 material with N^4^-(3-(triethoxysilyl) propyl) pyrimidine -2,4,6-triamine compound and was tested in the benzy lalcohol oxidation with TBHP as the oxidant under solventless conditions, when a high selectivity (97%) to benzaldehyde was achieved for a maximum conversion of 62% [76]. Heterogeneous Mn-nano-catalysts were synthesized and covalently anchored on a modified nanoscale SiO_2_/Al_2_O_3_ and were highly selective catalysts for the oxidation of cyclohexene (84% conversion and 95% selectivity for 2-cyclohexen-1-one) and benzyl alcohol (76.1% conversion, 100% selectivity for benzaldehyde) without the need for any solvent and using TBHP as the oxidant [77].

### 3.5. Catalyst Integrity and Leaching

The copper and manganese leaching out from the solid catalysts during the reaction were tested. For this purpose, the hot filtration test was conducted for the cyclohexene oxidation with H_2_O_2_ by using the catalysts with a good oxidative activity (SBA-15-NH_2_MetMn and SBA-15-NH_2_-MetCu). After the filtration of the reaction mixture at 303 K, the residual activity of the filtrate was further studied in the same reaction conditions.

The catalyst filtration was conducted at the reaction temperature in order to avoid possible re-coordination or precipitation of the metallic ions upon cooling. We found that after the hot filtration, there was no progress of reaction for the copper complex and a 5% conversion for the manganese complex sample. Furthermore, no copper was evidenced by AAS in the reaction products, and a very low manganese content, confirming that during the catalytic tests, there was no Cu leaching, and only a very slight content for Mn. The reusability of SBA-15-MetCu and SBA-15-NH_2_-MetMn catalysts was examined over five consecutive catalytic runs under identical reaction conditions (Figure 15). The loss of conversion was insignificant for Cu complex and significant between the first and second runs for the Mn complex due to the leaching of physically adsorbed catalytic sites. However, after the second run, no such loss was noted. Furthermore, Cu complexes were reused in five consecutive runs, and the catalytic performances have not decreased significantly.

## 4. Conclusions

A series of new Cu(II) and Mn(II) complexes with Schiff base ligands derived from 2-furylmethylketone, 2-furaldehyde, and 2-hydroxyacetophenone have been synthesized in situ into different mesoporous silica supports (MCM-41, SBA-15, MCM-48). The functionalization of silica in two steps preserved the structure, texture, and morphology of the supports with an ordered porous structure. The spectroscopic measurements sustain the presence of silica support surfaces of the metal complexes. EPR characterization shows that the interaction of Mn ions with ligands is weaker, and bonds are more ionic than for Cu complexes. The effects of ligand type and silica supports were evidenced for Cu complexes, and a good correlation was evidenced between catalytic properties and EPR results.

The immobilized Cu(II) and Mn(II) complexes were active catalysts for the oxidation of cyclohexene or aromatic and aliphatic alcohols (benzyl alcohol, 2-methylpropan-1-ol, 1-buten-3-ol). Comparing the effect of silica supports with catalytic activity was evidenced by the higher performances of the metal complexes supported on MCM-41. Higher catalytic activity was obtained for Mn compounds, especially in the oxidation of cyclohexene, even if the M/L ratio and the metal content were lower, probably due to a greater dispersion of manganese on the support surface. The highest selectivity was obtained for aldehydes in the oxidation of benzyl alcohol and 2-methylpropan-1-ol, respectively, and allylic oxidation products for cyclohexene and 1-butene-3-ol.

The obtained results pave the way for the development of highly effective heterogeneous catalysts, obtained by the in situ immobilization of metal complexes into mesoporous silica matrices, for the efficient oxidation of alkene and alcohol substrates.

## Figures and Tables

**Table 1:** Textural and structural properties of mesoporous silica modified with aminopropyl groups.

Sample	a_0_^a^ (nm)	Surface Area m^2^/g	Mesopore Volume (mL/g)	Pore Diameter ^b^ (nm)	V_BdB_^c^ (nm)	E ^d^ (nm)
SBA-15	11.3	600	1.1	7.8	10	3.5
SBA-15-NH_2_	11.4	388	0.8	7.2	9.2	4.2
MCM-41	4.8	902	0.7	3.5	3.8	1.3
MCM-41-NH_2_	4.5	683	0.4	2.4	3.5	2.1
MCM-48	9.6	1355	1.1	3.2	3.8	1.1
MCM-48-NH_2_	9.4	1033	0.5	3.0	3.2	1.7

^a^ Hexagonal: $a_{0}=2d_{100}\sqrt{3}$, Cubic: $a_{0}=d_{211}\sqrt{6}$; ^b^ D ≅ 4V/A; ^c^ mesopore volume by BdB method; ^d^ wall thickness= a_0_–D.

**Table 2:** Chemical composition of the metal complexes and their immobilization yield.

Sample	Ligand ^a^ mmol/g	Y_imm._ %	Metal mmol/g	Sample	Ligand ^a^ mmol/g	Y_imm._ %	Metal mmol/g
SBA-15-MetCu	0.96	48.00	0.33	MCM-41-HydCu	0.89	46.35	0.14
SBA-15-FurCu	1.25	62.50	0.32	MCM-41-MetMn	1.04	54.17	0.11
SBA-15 HydCu	0.80	41.02	0.17	MCM-41-FurMn	0.88	45.83	0.10
SBA-15-FurMn	0.78	39.00	0.12	MCM-48-MetCu	1.35	56.25	0.44
SBA-15-MetMn	0.95	47.50	0.11	MCM-48-FurCu	1.33	55.42	0.42
MCM-41-MetCu	1.51	62.92	0.32	MCM-48-FurMn	0.70	29.17	0.15
MCM-41-FurCu	1.71	89.63	0.30	MCM-48- MetMn	0.78	34.51	0.18

^a^ Determined by elemental analysis.

**Table 3:** The *g* and hyperfine coupling constants obtained by simulation of EPR experimental spectra of copper and manganese complexes.

Complex	EPR Parameters
MCM-41-NH_2_-Fur Cu	g_┴_ = 2.05, g_║_ = 2.34; G = 6.8; α^2^ = 0.40
MCM-41-NH_2_-MetCu	g_┴_ = 2.07, g_║_ = 2.223; G = 3.2; α^2^ = 0.30
MCM-48-NH_2_-HydCu	g_1_ = 2.09, g_2_ = 2.025, g_3_ = 2.27; G = 10.8; α^2^ = 0.23 A_1_ = 9.55 G, A_2_ = 9.35 G, A_3_ = 197.95 G
SBA-15-NH_2_-MetCu	g_1_ = 2.068, g_2_ = 2.043, g_3_ = 2.286; G = 6.7; α^2^ = 0.22 A_1_ = 3.54 G, A_2_ = 3.57 G, A_3_ = 178.35 G
SBA-15-NH_2_-FurCu	g_1_ = 2.054, g_2_ = 2.041, g_3_ = 2.306; G = 7.5; α^2^ = 0.24 A_1_ = 3.58 G, A_2_ = 3.59 G, A_3_ = 160.59 G
MCM-48-NH_2_-MetMn	g_1_ = 2.001, g_2_ = 2.007, g_3_ = 1.990 A_1_ = 105.92 G, A_2_ = 81.02 G, A_3_ = 92.24 G
MCM-41-NH_2_-FurMn	g_1_ = 2.000, g_2_ = 2.003, g_3_ = 1.990 A_1_ = 91.49 G, A_2_ = 89.21 G, A_3_ = 97.05 G
MCM-41-NH_2_-HydMn	g_1_ = 1.997, g_2_ = 2.045, g_3_ = 1.984 A_1_ = 105.13 G, A_2_ = 66.44 G, A_3_ = 86.00 G

**Table 4:** Catalytic performance of Cu(II) and Mn(II) complexes immobilized on SBA-15 mesoporous support in oxidation of cyclohexene.

Sample	CH Conversion (%)	TOF, (h^−1^)	Selectivity * (%)			
			I	II	III	IV
SBA-15-NH_2_-MetMn	91.5	164.2	70	16	13.7	0.3
SBA-15-NH_2_-MetCu	82.9	49.6	73	18	9.0	1.0
SBA-15-NH_2_-FurCu	76.8	47.4	77	12	10.0	1.0
SBA-15-NH_2_-FurMn	90.7	149.2	65	20	15.6	0.6

* Products formed: 2-cyclohexen-1-one (I), 2-cyclohexen-1-ol (II), 1,2-cyclohexendiol (III), and cyclohexene oxide (IV).

## Data Availability

The data presented in this study are available on request from the corresponding author.
